# Effects of aerobic training and vitamin D supplementation on glycemic indices and adipose tissue gene expression in type 2 diabetic rats

**DOI:** 10.1038/s41598-023-37489-z

**Published:** 2023-06-23

**Authors:** Kimya Khaledi, Rastegar Hoseini, Ahmad Gharzi

**Affiliations:** 1 Department of Exercise Physiology, Faculty of Sport Sciences, Razi University, P.O. Box. 6714414971, Kermanshah, Iran; 2 Department of Biology, Faculty of Science, Razi University, Kermanshah, Iran

**Keywords:** Metabolic disorders, Endocrinology

## Abstract

Type 2 diabetes mellitus (T2DM) is a progressive metabolic disorder mainly caused by overweight and obesity that accumulates pro-inflammatory factors in adipose tissue. Studies have confirmed the efficacy of exercise and vitamin D supplementation in preventing, controlling, and treating diabetes. While, reduced physical activity and vitamin D deficiency are related to increased adiposity, blood glucose level, insulin concentration, and insulin resistance. This study purposed to investigate the effect of 8-week aerobic training with vitamin D supplementation on the expression of AMPK, PGC-1α, and UCP-1 genes expression in the visceral adipose tissue of obese rats with T2DM. In this experimental study, fifty male Wistar rats were divided into 5 groups (n = 10): aerobic training and vitamin D supplementation (AT + Vit D), aerobic training (5 days/week for 8 weeks; AT), vitamin D supplementation (Vit D), diabetic control (C) and NC (Non-Diabetic Control). AT + Vit D and AT groups practiced an 8-week aerobic training, 5 days a week. Vit D and AT + Vit D groups receive 5000 IU of vitamin D by injection once a week while AT and C received sesame oil. After blood sampling, visceral fat was taken to measure AMPK, PGC-1α, and UCP1 gene expression. Data were statistically analyzed by One-way ANOVA and paired sample *t*-test at a significance level of p < 0.05. Based on our results BW, BMI, WC, visceral fat, insulin, glucose, and HOMA-IR were significantly lower in the AT + Vit D, AT, and Vit D groups compared with the C group (p < 0.01). Furthermore, AT + Vit D, AT, and Vit D upregulated AMPK, PGC-1α, and UCP1 gene expression compared to the C. Based on the results compared to AT and Vit D, AT + Vit D significantly upregulated AMPK (p = 0.004; p = 0.001), PGC-1α (p = 0.010; p = 0.001), and UCP1 (p = 0.032; p = 0.001) gene expression, respectively. Also, AT induced more significant upregulations in the AMPK (p = 0.001), PGC-1α (p = 0.001), and UCP1 gene expression (p = 0.001) than Vit D. Vitamin D supplementation enhanced the beneficial effects of aerobic training on BW, BMI, WC, visceral fat, insulin, glucose, and HOMA-IR in diabetic rats. We also observed that separate AT or Vit D upregulated the gene expression of AMPK, PGC-1α, and UCP1 however, combined AT + Vit D upregulated AMPK, PGC-1α, and UCP1 more significantly. These results suggested that combining aerobic training and vitamin D supplementation exerted incremental effects on the gene expressions related to adipose tissue in animal models of diabetes.

## Introduction

Type 2 diabetes mellitus (T2DM) is a progressive metabolic disease with a growing prevalence. Studies have shown 382 million cases in 2013 globally, which is expected to reach 592 million by 2035^1^. This multi-risk-factored disease is caused by defects in the function and secretion of insulin leading to increased blood glucose and B cell destruction consequently^2^. Although several lines of action have been considered to manage T2DM, there is an increasing need to find better approaches to control T2DM more effectively^3,4^. Recently, studies have focused on investigating the biological pathways involved in maintaining energy homeostasis to reduce the adverse effects of insulin resistance and metabolic disorders resulting from excessive food consumption and obesity^5^. For instance, AMP-Activated Protein Kinase (AMPK) is one of these biological pathways that stimulate FFA oxidation, inhibit FFA synthesis and lipolysis in adipose tissue, and signal the hypothalamus to increase food intake^6^. In addition, T2DM is associated with the reduced conversion of White Adipose Tissue (WAT) to brown adipose tissue, probably due to decreased expression and activation of specific browning genes such as Uncoupling Protein 1 (UCP1) and Peroxisome Proliferator-Activated Receptor Gamma Coactivator 1-Alpha (PGC-1α)^7^. The results of studies have shown that aerobic training is a powerful non-pharmacological way to confront obesity-related diseases, especially T2DM^8,9^, which has been an important lifestyle alteration to manage T2DM in recent years^10,11^. Cao et al. investigated the role of exercise on AMPK showing both acute and chronic exercises increased the phosphorylation and expression of AMPK and its downstream signaling pathway (e.g. PI3K) in diabetic rats^12^. Meanwhile, the results of Takekoshi et al. research showed that long-term aerobic training on a treadmill induced an increase in AMPK activity by about 1.30-fold compared with that of sedentary controls in visceral adipose tissue but has no effect on AMPK mRNA levels in subcutaneous adipose tissue^13^. Kang et al. also showed that long-term swimming training increases the expression of PGC-1α in obese mice^14^. Handschin and Spiegelman reported that 3-week moderate-intensity endurance training on a treadmill increases the expression of UCP1 mRNA in subcutaneous adipose tissue and visceral adipose tissue of rats leading to increased expression of PGC-1α^15^. However, some studies observed decreased expression of PGC-1α, and UCP1 while some reported no change in PGC-1α following exercise^16,17^. On the other hand, various studies have investigated the effect of supplements on patients with T2DM; Among them, studies have associated vitamin D deficiency with changes in insulin and blood glucose, as well as the sensitivity of specific tissues to insulin and consequently the risk of developing T2DM^18,19^. Also, since vitamin D is adipose-soluble and is stored in large amounts in adipose tissue, the serum level of vitamin D is probably lower in obese individuals^20,21^. A recent study has shown that vitamin D supplementation increased the effectiveness of aerobic training in reducing visceral fat, insulin, and blood glucose in Rats with metabolic syndrome and concluded that adequate vitamin D level is essential to attain the metabolic effects of physical activity^22^. Also, Cordeiro et al. reported reduced body weight gain and abdominal adiposity in obese rats following 30 days of vitamin D supplementation in western diet-fed obese rats^23^. Vitamin D alters insulin sensitivity in two main ways; regulating insulin receptor gene expression, which can be related to insulin resistance, and mediating calcium metabolism, thus increasing glucose transporter bioactivity^21^.

Accordingly, we hypothesized the possibility of the effect of vitamin D on the secretion and sensitivity of insulin through activating AMPK, UCP-1, and PGC-1α gene expression in visceral adipose tissue.

Although it is well established that separate aerobic training and vitamin D supplementation improve glucose tolerance and diabetes-related factors in skeletal muscle, the long-term effect of combined aerobic training with vitamin D supplementation on the expression of AMPK, PGC-1α, and UCP-1 genes in the visceral adipose tissue remains unknown. Considering the complications of T2DM and the increasing prevalence, this study aimed to investigate the effect of 8-week aerobic training with vitamin D supplementation on AMPK, PGC-1α, and UCP-1 gene expression in the visceral adipose tissue of obese rats with T2DM.

## Methods

### Ethical approval

All the procedures of the present study were submitted and approved by the Committee of Ethics and Research on the Use of Animals of the Razi University of Kermanshah (IR.RAZI.REC.1401.013) and experiments were performed following the specific Iranian laws on the Bioethics in Experiments with Animals (nº 11.794/2008) and complied with the ARRIVE guidelines for the Care and Use of Laboratory Animals.

### Animals

In this experimental study, fifty 4–5-week-old male Wistar rats weighing 180 ± 10 g were selected and divided into two main groups; diabetic (n = 40 rats) and Non-Diabetic Control (NC; n = 10 rats) groups. After inducing diabetes through intraperitoneal streptozotocin (STZ) and nicotinamide with a High-Fat Diet (HFD), diabetic rats were randomly assigned to 4 groups (10 in each group); aerobic training + vitamin D supplementation (AT + Vit D), aerobic training (AT), vitamin D supplementation (Vit D), control (C). AT and AT + Vit D groups practiced an 8-week aerobic training, 5 days a week. Vit D and AT + Vit D groups receive 5000 IU of vitamin D by injection once a week. While, AT and C were injected with sesame oil instead. This placebo-controlled randomized and single-blinded study was registered in the Iranian registry of clinical trials at http://www.irct.ir: number, IRCT20220510054809N1. Also, the Research Ethics Committee of Razi University approved and supervised the study design and conduction (no. IR.RAZI.REC.1401.013), Iran. Animal care, maintenance and sacrifice have been conducted according to the Danish “Animal Welfare Act” (LBK 1343 of 04/12/2007).

### Obesity induction method

Two weeks prior to the commencement of the interventional program rats were acclimatized to the new environment. Then, male rats were fed high-fat diet to increase weight in the form of pellets (purchased from Beh-Parvar Company); a mixture of standard mouse food powder (365 mg/kg), sheep fat (310 mg/kg), mixed vitamins and minerals (60 mg/kg), dl-methionine (3 mg/kg), yeast powder (1 mg/kg), and chloride sodium (1 mg/kg). Animals were kept in transparent polycarbonate cages with a length of 30 cm, and width and height of 15 cm. Animals were maintained under a 12-h light/dark cycle, temperature of 25 ± 2 °C, and 45–55% humidity. All animals had free access to special food and water in a 500 ml bottles^16,24^; the ethical principles of working with laboratory animals were considered in the present study.

### Diabetes induction method

After increasing weight (more than 300 g) following 2 weeks, 60 mg/kg BW streptozotocin (dissolved in 0.1 M citrate buffer with pH = 4.5) and then, after 15 min, 110 mg/kg BW of nicotinamide were intraperitoneally injected to induce diabetes in male Wister rats. To ensure the induction of T2DM, blood sugar was measured 2 weeks after the injection with the help of a glucometer and blood samples of the lateral tail vein after immersing in 42 °C water for 40–50 s; Blood sugar above 200 mg/l was considered as an indicator of diabetes^25^. Animals received no insulin treatment during the study.

### Intervention programs

#### Aerobic training protocol

**Figure 1:**
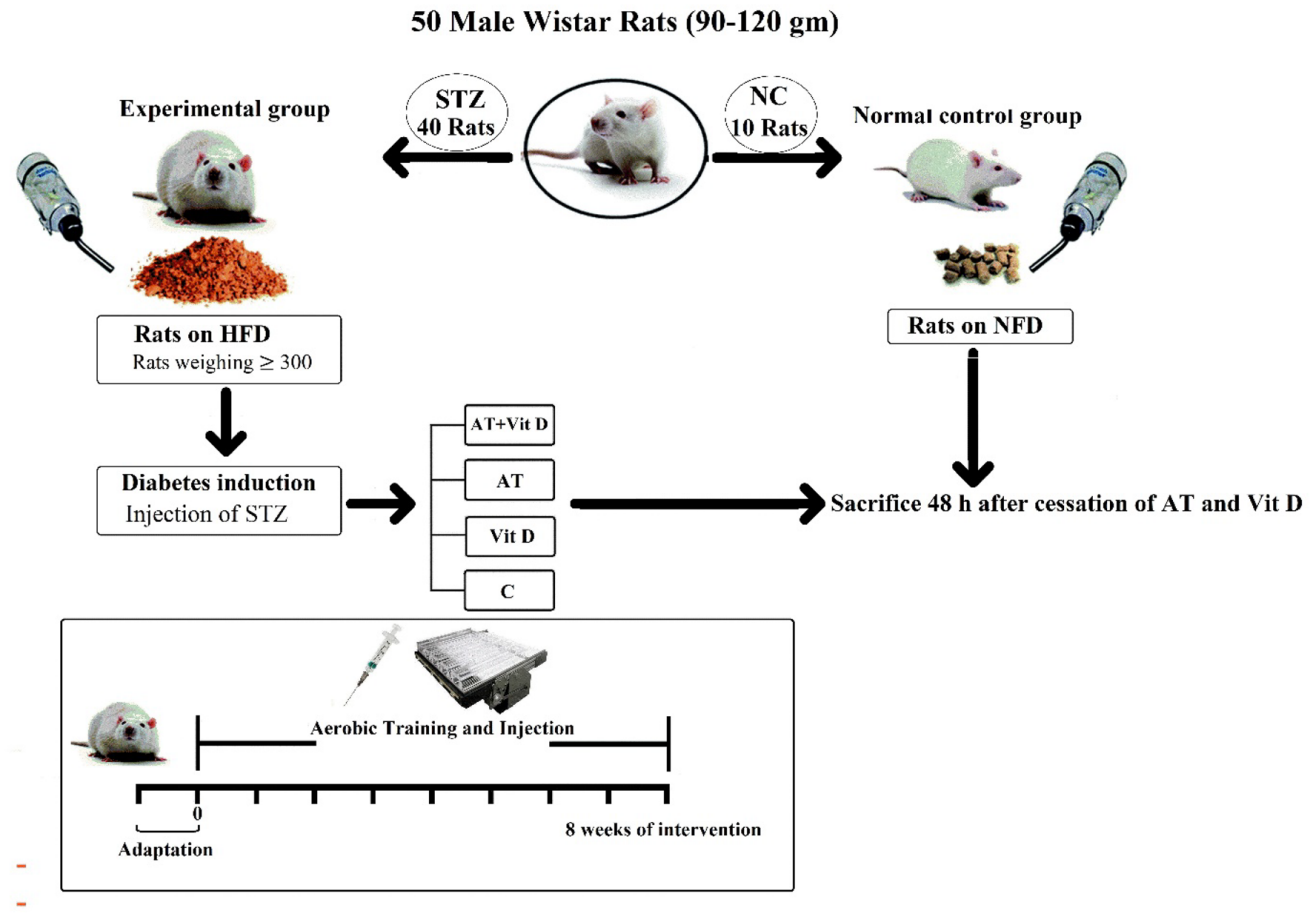
Flow chart of the study. *AT* + *Vit D* Aerobic Training + Vitamin D Supplement, *AT* Aerobic Training, *Vit D* Vitamin D Supplement, *C* Control, *NC* Non-Diabetic Control.

After the induction of diabetes, the rats were placed in 4 groups of 10 randomly and also through weight equalization. One week before the start of the main aerobic training sessions rats ran on the treadmill for 5 min at a speed of 8–10 m/min, 5 days a week to get familiar with the treadmill. The aerobic training program consisted of 8 weeks of running on a zero-incline treadmill, at 15–25 m/min, for 30–60 min, 5 days a week. In the first week, rats performed 30 min of aerobic training on a treadmill at a speed of 15 m/min. Then, the exercise intensity and duration increased gradually to reach 25 m/min and 60 min in the 8th week. The training intensity was equal to 50–60% of VO_2max_^26^. Animals also warmed up and cooled down for 10 min at 5 m/min before and after the aerobic training. To stimulate stopped rats to run, a combination of sound (hitting the wall of the rotating tape) and low voltage electric stimulations were used. In the first week, the low voltage electric stimulus was used along with the sound stimulus. Then, only the sound stimulus was used considering the ethical guidelines of working with laboratory animals^16,24^ (Fig. 1).

### Vitamin D supplementation

In the present study, Vit D and AT + Vit D groups received 5000 international units (IU) of vitamin D per week by single-dose injection. AT and C were injected with sesame oil instead of vitamin D. Vitamin D serum concentration was measured using a vitamin D enzyme-linked immunosorbent assay (ELISA) rat kit (immune diagnostics system Ltd, Boldon, UK; with intraassay coefficient of variation = 1.63%, and sensitivity of method = 1.33 mg/dL)^22^.

### Food intake, bodyweight, and body mass index

All animals were weighed weekly, between 09:00 and 11:30 a.m. using a scale. Body length (nose-to-anus) and Body Mass Index (BMI) were calculated. Food Intake (FI) was measured by subtracting the weight of the uneaten food from the total equal amount of food (20 g/d) given in each cage.

### Tissue and blood sampling

In order to eliminate the acute effects of training and uncontrollable variables caused by the training program, 48 h after the last training session, the animals were anesthetized (46, 15) by intraperitoneal injection of a combination of ketamine (70 mg/kg BW) and xylazine (3–5 mg/kg BW) considering the ethical principles. The blood sample was taken from the vena cava; The plasma concentrations of glucose and insulin were measured by the Hitachi Auto Analyzer (type 7170; Hitachi Electronics, Hitachi, Japan). The Homeostatic Model Assessment for Insulin Resistance (HOMA-IR) was calculated using the concentration of glucose and insulin as follows:

$$ {\text{HOMA - IR}} = \left( {{\text{glucose}}\;{\text{concentration}} \times {\text{insulin}}\;{\text{concentration}}} \right) \div 22.5 $$

Visceral adipose tissue was immediately separated and weighed. After washing with saline, to prevent RNA degradation, it was placed in tubes containing RNA Later, transferred to liquid nitrogen, and then stored in a refrigerator at − 80 °C for further measurements.

### RNA extraction/real-time PCR

RNA isolation from 20 mg of adipose tissue was performed using the combined protocol of TRI Reagent^®^ (MRC Inc., US) and miRNeasy methods (Viragene, Iran). First, the frozen tissue was homogenized as recommended by the manufacturer using a gentleMACS™ Octo Dissociator system, M tubes (Viragene, Iran), and the RNA_02 program in 2 ml of TRI Reagent^®^ buffer. After incubating at room temperature for 5 min, the samples were centrifuged at 12,000*g* at 4 °C for 10 min. The desired layer was piped into a 1.5 ml tube, mixed with 400 μl of chloroform, and centrifuged at 12,000*g* at 4 °C for 30 min after keeping the sample for 3 min at room temperature. Then the upper layer was mixed with ethanol by inverting the tube several times and loaded on the miRNeasy spin column (Viragene, Iran) performing the manufacturer’s protocol. Finally, RNA was eluted in 30 μl of RNAse-free water. Reverse transcription into cDNA was performed using 1 μg of total RNA with a Prime Script RT reagent kit (Viragene, Iran). Quantitative RT-PCR was performed using TB Green Premix Ex-Taq II (TaKaRa, Dalian, China). Beta-2-Microglobulin (B2M) was used as the reference gene to measure relative gene expression. The results were evaluated by using 2^−ΔΔCt^ comparative method and Light Cycler SW1.1 software. The sequence of the primers is shown in Table 1. The Viragene kit, Iran was used to measure AMPK gene expression with a sensitivity of 0.021; to measure PGC-1α gene expression Viragene mini kit50, Iran was used with a sensitivity of 0.06; And to measure UCP-1 gene expression, Viragene kit, Iran was used with a sensitivity of 0.06.

**Table 1:** The forward and reverse primer sequences.

Genes	Primers	
B2m	Forward	GTCTCGCTCCGTGGCCTTA
	Reverse	TGGAGTACGCTGGATAGCCTC
PGC-1 α	Forward	AGCCTCTTTGCCCAGATCTT
	Reverse	GGCAATCCGTCTTCATCCAC
AMPK	Forward	AAGCCGACCCAATGACATCA
	Reverse	CTTCCTTCGTACACGCAAAT
UCP-1	Forward	GTGAAGGTCAGAATGCAAGC
	Reverse	AGGGCCCCCTTCATGAGGTC

### Statistical analysis

Data were analyzed using Statistical Package for Social Sciences (SPSS, Inc., Chicago, IL, USA) version 26. To check the normality of the distribution in the continuous variables the Shapiro–Wilk test was used. The paired sample *t*-test was used to do within-group comparisons. Also, one-way ANOVA and Tukey's post-hoc to compare between-groups. The significance level was set at *p* < 0.05.

### Ethical approval

This study was approved by the Ethics Committee of Razi University of Kermanshah (IR.RAZI.REC.1401.013) and was registered in the Iranian Clinical Trial Registration Center (code: IRCT20220510054809N1 on 27/06/2022).

## Results

Mean body weight, BMI, and food intake (FI) are shown in Table 2. There was a significant difference between groups in mean BW, BMI, and FI after 8-week of intervention; BW, BMI, and FI reduced significantly in the AT + Vit D, AT, and Vit D groups at the end of the study compared with the beginning (p < 0.01). The highest reduction in BW, BMI, and FI was in the AT + Vit D group, whereas mean BW, BMI, and FI increased significantly in the C and NC groups.

**Table 2:** Comparison of mean ± SD of body weight, BMI, FI, and WC before and after intervention.

Variables	AT + Vit D (n = 10)	AT (n = 10)	Vit D (n = 10)	C (n = 10)	NC (n = 10)	p-value ^a^
Body weight (g)						
Before	305.70 ± 2.90	308.10 ± 2.80	303.80 ± 2.78	304.70 ± 1.88	207.60 ± 3.80	
After	250.50 ± 5.79	263.80 ± 2.78	286 ± 3.19	322.90 ± 3.60	213.50 ± 4.35	
p^†^	0.001*	0.001*	0.001*	0.001*	0.009*	
Δ	− 55.20 ± 2.89^µ€αβ^	− 44.30 ± 0.02^€αβ^	− 17.80 ± 0.41^αβ^	− 18.20 ± 1.72^β^	5.90 ± 0.55	0.001^¥^
BMI (kg/m^2^)						
Before	0.74 ± 0.033	0.76 ± 0.019	0.77 ± 0.021	0.76 ± 0.030	0.52 ± 0.016	
After	0.61 ± 0.015	0.65 ± 0.011	0.71 ± 0.026	0.80 ± 0.016	0.53 ± 0.014	
p^†^	0.001*	0.001*	0.005*	0.006*	0.013*	
Δ	− 0.13 ± 0.018^µ€αβ^	− 0.11 ± 0.008^€αβ^	− 0.06 ± 0.005^αβ^	0.04 ± 0.014	0.01 ± 0.002	0.011^¥^
FI (g/day)						
Before	15.17 ± 0.026	15.30 ± 0.029	15.14 ± 0.018	15.18 ± 0.024	14.64 ± 0.048	
After	15.11 ± 0.024	15.23 ± 0.033	15.11 ± 0.025	15.61 ± 0.045	14.70 ± 0.030	
p^†^	0.002*	0.002*	0.002*	0.001*	0.023*	
Δ	− 0.06 ± 0.02^αβ^	− 0.07 ± 0.04^αβ^	− 0.03 ± 0.07^αβ^	0.7 ± 0.01^β^	0.06 ± 0.018	0.002^¥^

*AT* + *Vit D* Aerobic Training + Vitamin D Supplement, *AT* Aerobic Training, *Vit D* Vitamin D Supplement, *C* Control, *NC* Non-Diabetic Control.

Data analysis was done by the analysis of one-way analysis of variance test followed by post hoc Tukey's test; p^†^: Statistical analysis was done by paired sample *t*-test.

*Significantly different in comparison pre and post within the groups.

^¥^Significantly different comparing Δ between groups.

^µ^Significantly different compared to AT.

^€^Significantly different compared to Vit D.

^α^Significantly different compared to C.

^β^Significantly different compared to Sham.

As Table 3 shows, there was a statistically significant difference between groups in visceral fat, insulin, glucose, and HOMA-IR; with the lowest level in AT + Vit D and the highest in the C group. While, the results show a statistically significant difference between groups in serum 25-hydroxyvitamin D; with the highest level in AT + Vit D and the lowest in the C group. Based on the results, the mean visceral fat, insulin, glucose, and HOMA-IR were significantly lower in the AT + Vit D, AT, and Vit D than in the C group. In addition, significant differences were observed in visceral fat, insulin, glucose, HOMA-IR, and serum 25-hydroxyvitamin D between the NC group and other groups (p < 0.05 for all three variables).

**Table 3:** Comparison of mean ± SD of visceral fat, insulin, glucose, HOMA-IR, and vitamin D after the intervention among the groups.

Variables	AT + Vit D (n = 10)	AT (n = 10)	Vit D (n = 10)	C (n = 10)	NC (n = 10)	p-value
Visceral fat	9.23 ± 0.026^D^	9.89 ± 0.031^C^	10.32 ± 0.049^B^	11.18 ± 0.033^A^	8.80 ± 0.045 ^E^	0.001^¥^
Insulin	2.59 ± 0.025^D^	2.82 ± 0.022^C^	2.96 ± 0.040^B^	3.24 ± 0.018^A^	1.19 ± 0.029^E^	0.001^¥^
Glucose	18.89 ± 0.028^D^	19.69 ± 0.021^C^	23.39 ± 0.034^B^	29.50 ± 0.023^A^	8.23 ± 0.018^E^	0.001^¥^
HOMA-IR	0.12 ± 0.0012^D^	0.13 ± 0.001^C^	0.17 ± 0.002^B^	0.23 ± 0.001^A^	0.024 ± 0.001^E^	0.001^¥^
Vit D (nmol/L)	120.20 ± 2.14^B^	108.50 ± 1.58^D^	116.60 ± 1.26^C^	86.60 ± 1.71^E^	129.20 ± 1.31^A^	0.001^¥^

*AT* + *Vit D* Aerobic Training + Vitamin D Supplement, *AT* Aerobic Training, *Vit D* Vitamin D Supplement, *C* Control, *NC* Non-Diabetic Control.

One-way ANOVA test followed by Tukey’s posthoc test.

Dissimilar letters represent a significant difference between the groups, the level of significance was set at 5% (p < 0.05).

^¥^Significantly different comparing Δ between groups.

**Figure 2:**
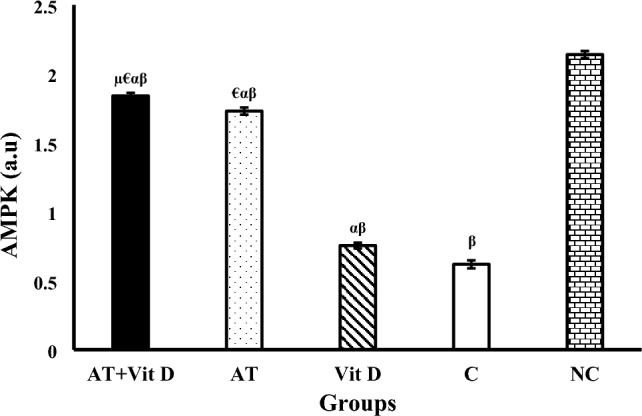
Comparison between mean ± SD of AMPK gene expression between groups. Values were calculated using a one-way analysis of variance followed by post hoc Tukey's test. *AT* + *Vit D* Aerobic Training + Vitamin D Supplement, *AT* Aerobic Training, *Vit D* Vitamin D Supplement, *C* Control, *NC* Non-Diabetic Control. ^µ^Significantly different compared to AT. ^€^Significantly different compared to Vit D. ^α^Significantly different compared to C. ^β^Significantly different compared to NC.

**Figure 3:**
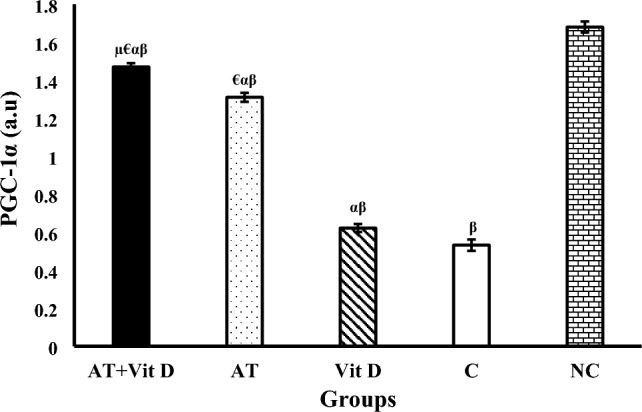
Comparison between mean ± SD of PGC-1α gene expression between groups. Values were calculated using a one-way analysis of variance followed by post hoc Tukey's test. *AT* + *Vit D* Aerobic Training + Vitamin D Supplement, *AT* Aerobic Training, *Vit D* Vitamin D Supplement, *C* Control, *NC* Non-Diabetic Control. ^µ^Significantly different compared to AT. ^€^Significantly different compared to Vit D. ^α^Significantly different compared to C. ^β^Significantly different compared to NC.

**Figure 4:**
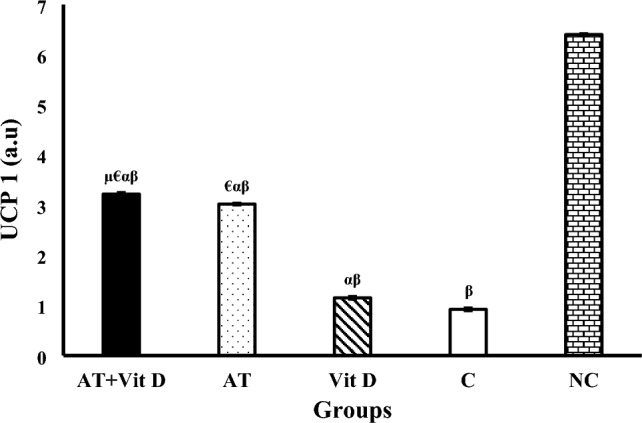
Comparison between mean ± SD of UCP1 gene expression between groups. Values were calculated using a one-way analysis of variance followed by post hoc Tukey's test. *AT* + *Vit D* Aerobic Training + Vitamin D Supplement, *AT* Aerobic Training, *Vit D* Vitamin D Supplement, *C* Control, *NC* Non-Diabetic Control. ^µ^Significantly different compared to AT. ^€^Significantly different compared to Vit D. ^α^Significantly different compared to C. ^β^Significantly different compared to NC.

The results of AMPK, PGC-1α, and UCP1 gene expression are shown in Figs. 2, 3, 4. There was a significant difference in AMPK, PGC-1α, and UCP1 gene expression between the diabetic and NC groups at the end of the intervention. One-way ANOVA showed a significant difference in Akt, PEPCK, and G6Pase gene expression between diabetic groups. Furthermore, AT + Vit D, AT, and Vit D upregulated AMPK, PGC-1α, and UCP1 compared to the C. Based on the results, AT + Vit D significantly upregulated AMPK (p = 0.004; p = 0.001), PGC-1α (p = 0.010; p = 0.001), and UCP1 (p = 0.032; p = 0.001) compared to AT and Vit D, respectively. Also, AT induced more significant upregulations in the AMPK (p = 0.001), PGC-1α (p = 0.001), and UCP1 gene expression (p = 0.001) than Vit D.

## Discussion

In the present study, we initially demonstrated that separate aerobic training and vitamin D improved body weight and serum parameters such as fasting blood glucose, insulin, and HOMA-IR. However, better results were observed when combining aerobic training and vitamin D. Our results were consistent with those of previous studies indicating the same improvements following aerobic training^27,28^. Also, studies showed that 8 weeks of Vitamin D supplementation reduced body weight, BMI, visceral fat, waist circumference, serum non-HDL cholesterol, and TG^29,30^. Vitamin D is a hormone that is synthesized in the skin and plays a vital role in maintaining optimal body function, particularly in the regulation of calcium homeostasis and bone health. The level of vitamin D in the body is influenced by various factors, including dietary intake, sun exposure, and physical activity^31^. The cellular and molecular mechanism of the observed differences in vitamin D values can be traced to the effect of exercise-induced changes on vitamin D metabolism^32^. During physical activity, there is an increase in the circulating levels of parathyroid hormone (PTH), a hormone that plays a crucial role in the regulation of calcium and phosphate homeostasis^33^. The increase in PTH levels stimulates the conversion of 25-hydroxyvitamin D [25(OH)D], the primary circulating form of vitamin D, to its active form, 1,25-dihydroxy vitamin D [1,25(OH)2D], in the kidneys^32,33^.

Moreover, exercise stimulates the increase in vitamin D receptor (VDR) expression in muscle cells as well as the synthesis of vitamin D binding protein (DBP) in the liver, which facilitates the transport and uptake of vitamin D into the muscles^34^. These changes ultimately result in an increase in the intracellular concentration of 1,25(OH)2D in the muscle fibers, which contributes to the beneficial effects of exercise on muscle health and function^27^.

However, the observed differences in vitamin D values may have been influenced by exercise-induced changes in vitamin D metabolism, as well as variations in initial levels. Understanding the cellular and molecular mechanisms of these factors can help researchers design more effective interventions to optimize vitamin D status and promote overall health. Therefore, it is crucial to control for these factors in any study examining the relationship between exercise and vitamin D status.

The molecular mechanisms of aerobic training in the improvement of diabetes might include the upregulation of insulin transporters in the cell membrane, reduction of adipokines, inflammatory and oxidative stress responses, and improvement of insulin signal transduction leading to higher insulin sensitivity. Another interesting point made in this study was that 8-week aerobic training increased gene expressions of AMPK in WAT of type 2 diabetes rats. In line with these results, studies have reported an increment in AMPK gene expression following acute and chronic exercises; Li et al. showed a simultaneous increase in AMPK and PGC-1α activity following chronic exercise training^35^. Park et al. observed a stimulatory role for short-term exercise on AMPK activation in visceral adipose tissue suggesting that aerobic training could stimulate basal AMPK activity in visceral adipose tissue^36^. Based on the results of the present study, alteration of AMPK mRNA levels in visceral adipose tissue could be involved in ameliorating T2DM following exercise adaptations. AMPK is involved in carbohydrate and fat metabolism, insulin signaling, and inflammation which are reduced in obesity^37^. Villena et al. reported increased body weight and adipose tissue mass in AMPK knockout mice which supports our results^38^. Besides the central role of AMPK as an energy sensor, AMPK has also been elucidated to have a paramount function in the browning of WAT and regulating the energy expenditure of brown/beige adipose tissue^39^. Our result also suggests that chronic exercise along with increased AMPK expression helps increase UCP-1 and PGC-1α gene expression. There are contradictory results in the literature regarding UCP1; Matteis et al. nominate exercise as a new physiological stimulus for brown adipose tissue activity but showed an insignificant increase in UCP-1^40^. While, Brandao et al. investigated physical training and UCP1 expression, in adipose tissue of obese women reporting reduced UCP1 mRNA expression in WAT following 8 weeks of exercise^41^. It seems that increased intracellular regulatory factors (such as PGC-1α) following aerobic training and vitamin D supplementation might have altered the UPC-1 expression in this study. Furthermore, studies showed that increased secretion of hormones (e.g. thyroid hormones^42^, norepinephrine^43^, and Irisin^44^) during aerobic training might upregulate the UCP-1 gene expression in adipose tissue through different mechanisms. Moreover, a wide spectrum of studies support the increment of PGC-1α genes following aerobic training in skeletal muscle of humans^45^ and visceral adipose tissue of obese rats^16^. However, the observed weight loss in this study might be due to the reduced food intake following aerobic training and vitamin D supplementation. According to body weight and food intake results obtained in this study, the most significant reduction was in the group with aerobic training combined with vitamin D. The mechanism by which an increase in the concentration of vitamin D in the body weight and food intake affects body composition is difficult to explain. However, some studies reported no effect from vitamin D on body weight change and energy expenditure and little improvement in cardiovascular risks with cholecalciferol^46,47^. In agreement with our study, Hoseini et al. reported that vitamin D supplementation combined with aerobic training significantly improves BW and FI in ovariectomized rats^22^. Also, the decreased appetite hormone, and increased AMPK gene expression, and PGC-1α/UCP-1 pathway following aerobic training might be among the possible mechanisms. In addition, a proposed model related to the relationship between vitamin D deficiency and more food intake is that the decrease in the concentration of calcidiol flow in the hypothalamus induces an increase in the set point of body weight. On the other hand, the appetite increases and the energy consumption decreases through activating the hypothalamic NPY/AgRP neurons and POMC/CART inhibiting.

Furthermore, the results of the present study showed that AMPK, PGC-1α, and UCP1 expression increased after 8 weeks of vitamin D supplementation. Recent evidence has demonstrated that vitamin D is a potential stimulating factor in increasing AMPK activity and gene expression^48,49^. Gauthier et al. observed low AMPK activation in inflamed adipose tissue of obese human participants^50^. Chang investigated the role of vitamin D in oxidative stress and mitochondrial changes suggesting that the observed potent inhibitory effect of 1,25(OH)2D on muscle oxidative stress and mitochondrial dynamics might be at least involved in the activation of AMPK^49^. A recent research also studied the effects of vitamin D supplementation on adipose tissue inflammation and NF-κB/AMPK activation in obese mice fed a high-fat diet indicating a significant increase in AMPK activity by 1.4-fold following 16-week supplementation of vitamin D^48^. Vitamin D alters the genetic responses by binding to the Vitamin D Nuclear Receptor (VDR) and producing the Vitamin D Response Element (VDRE) which modifies AMPK, PGC-1α and UCP1 gene expression in WAT probably by binding to activating factors (COAC) or repressing factors (CORE)^51^. Additionally, vitamin D is believed to regulate lipolysis dependent on its localization; stimulating lipogenesis in subcutaneous tissue but inhibiting it in visceral adipose tissue^52^. Vitamin D supplementation was demonstrated to increase the expression of genes such as PGC-1α and PDK4 and thereby enhance the oxidation of fatty acids^53^. Vitamin D has long been known as a hormonal regulator of the cAMP signaling pathway^54^. In turn, cAMP activates cAMP-dependent PKA, the endocrine system, and AMPK activity^55^ which phosphorylates and activates lipolysis enzymes and leads to cascades of phosphorylation that stimulate the activity of PGC1α, PPARα, and UCP1^56,57^. The results also showed a significant increase in the gene expression of AMPK, PGC-1α, and UCP1 in WAT in AT + Vit D compared to the other three groups (Vit D, AT, and control). However, the main mechanism for combined AT + Vit D is not elucidated, it might be a better approach in inducing AMPK, PGC-1α, and UCP1 expression in WAT of T2DM rats.

### Strength and limitations of the study

Our study had several strengths using a randomized, placebo-controlled trial with no dropouts and evaluating the gene expression alterations in animal model of diabetes. However, it is premature to conclude a definitive answer. Also, not evaluating the levels of vitamin D before and after the induction of diabetes or at the beginning of the study and other important transcriptional and post-transcriptional factors were among the limitations of the present study.

## Conclusion

Vitamin D supplementation enhanced the beneficial effects of aerobic training on BW, BMI, WC, visceral fat, insulin, glucose, and HOMA-IR in diabetic rats. We also observed that separate AT or Vit D upregulated the gene expression of AMPK, PGC-1α, and UCP1 however, combined AT + Vit D upregulated AMPK, PGC-1α, and UCP1 more significantly. These results suggested that combining aerobic training and vitamin D supplementation exerted incremental effects on the gene expressions related to adipose tissue in animal models of diabetes.

## Data Availability

The datasets generated and analysed during the current study are not publicly available due to ongoing data analysis but are available from the corresponding author on reasonable request.

## Abbreviations

AT: Aerobic training
Vit D: Vitamin D
T2DM: Type 2 diabetes mellitus
AMPK: AMP-activated protein kinase
WAT: White adipose tissue
UCP1: Uncoupling protein 1
PGC-1α: Peroxisome proliferator-activated receptor gamma coactivator 1-alpha
VDR: Vitamin D nuclear receptor
VDRE: Vitamin D response element
POMC: Pro-opiomelanocortin
CART: Cocaine- and amphetamine-regulated transcript
